# Correction: MiR-124 acts as a target for Alzheimer’s disease by regulating BACE1

**DOI:** 10.18632/oncotarget.25461

**Published:** 2018-05-15

**Authors:** Fengmao An, Guohua Gong, Yu Wang, Ming Bian, Lijun Yu, Chengxi Wei

**Affiliations:** ^1^ Medicinal Chemistry and Pharmacology Institute, Inner Mongolia University for The Nationalities, Tongliao, Inner Mongolia, P.R. China; ^2^ Inner Mongolia Key Laboratory of Mongolian Medicine Pharmacology for Cardio-Cerebral Vascular System, Tongliao, Inner Mongolia, P.R. China; ^3^ First Clinical Medical of Inner Mongolia University for Nationalities, Tongliao, Inner Mongolia, P.R. China

**This article has been corrected:** The correct Materials and Methods and Figure 2 are given below: The authors declare that these corrections do not change the results or conclusions of this paper.

**Luciferase reporting assay**

The 3’ UTR of BACE1 and the CMV promoter were amplified from human chromosomal DNA and pcDNA3.1 (+) and cloned into the pGL3-luciferase basic vector (Promega, Madison, WI, USA). Sequences of primers and cloning strategy are available on request. For the luciferase assays, 50 nM of miR-124 mimics or scrambled RNA were co-transfected with the reporter vector and the Renilla control vector (Promega, Madison, WI, USA) into the HEK293 cells by Lipofectamine 2000 (Invitrogen, Carlsbad, CA, USA). 24 h post transfection, the measurements were performed using the Dual luciferase re-porter assay kit (Promega, Madison, WI, USA). Or the HEK293 cells post the transfection for 24 h was lyzed for western blot analysis.

**Figure 2:**
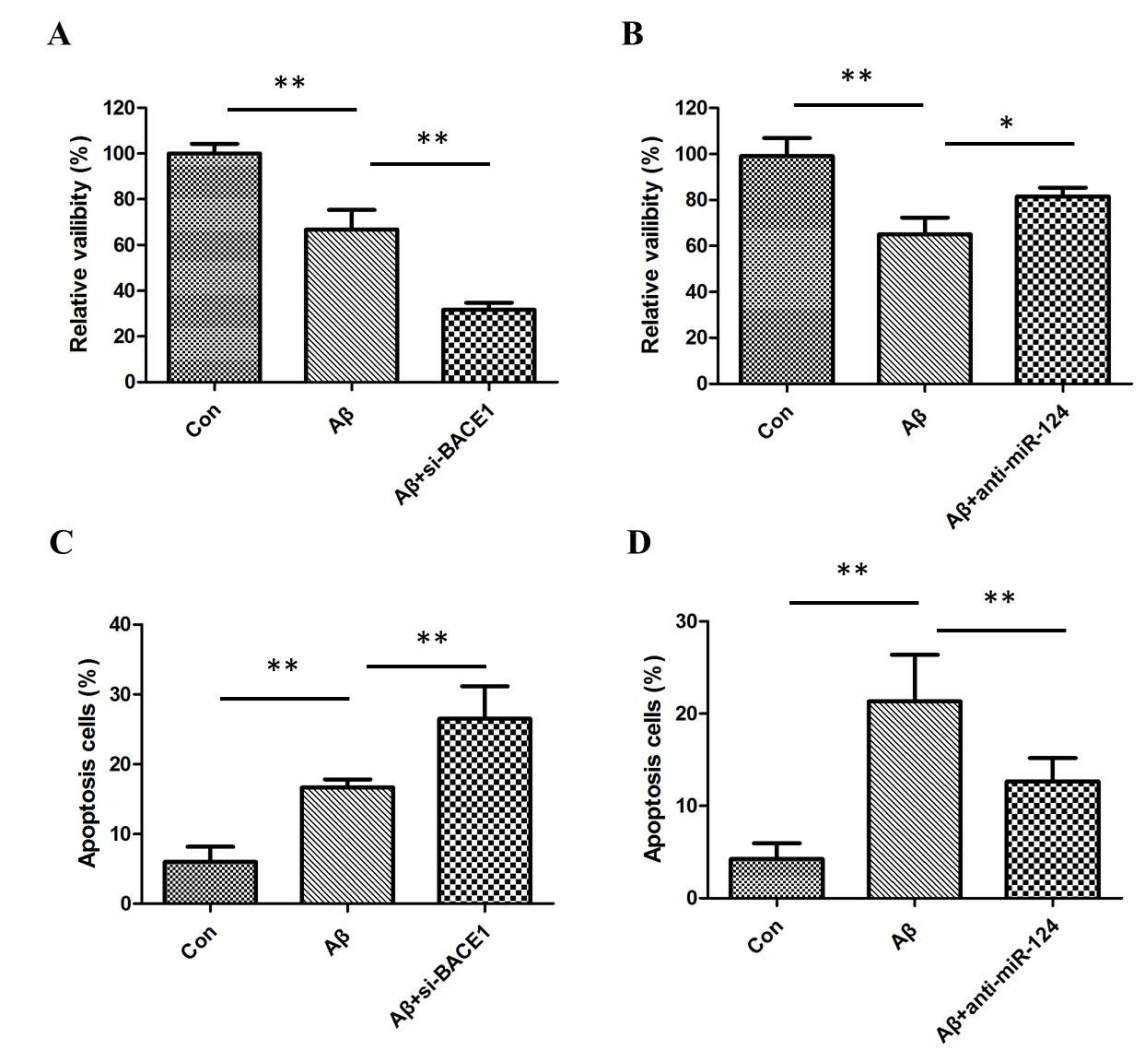
(**A**) MTT assay results showed that Aβ inhibited the viability of SH-SY5Y cells and downregulation of BACE1 enhanced the inhibitory effects of Aβ; (**B**) downregulation of miR-124 relieved Aβ-induced viability inhibition of SH-SY5Y cells; (**C**) flow cytometric analysis results showed that Aβ-induced apoptosis of SH-SY5Y cells and downregulation of BACE1 enhanced the induced effects of Aβ; (**D**) downregulation of miR-124 decreased apoptosis of SH-SY5Y cells in the presence of Aβ.

Original article: Oncotarget. 2017; 8:114065-114071. https://doi.org/10.18632/oncotarget.23119

